# Evaluation and comparison of computational tools for RNA-seq isoform quantification

**DOI:** 10.1186/s12864-017-4002-1

**Published:** 2017-08-07

**Authors:** Chi Zhang, Baohong Zhang, Lih-Ling Lin, Shanrong Zhao

**Affiliations:** 1 Early Clinical Development, Pfizer Worldwide R&D, Cambridge, MA 02139 USA; 2 Inflammation and Immunology RU, Pfizer Worldwide R&D, Cambridge, MA 02139 USA

**Keywords:** RNA-seq, Quantification, Isoform, Data analysis, RSEM, Salmon, Salfish, Kallisto

## Abstract

**Background:**

Alternatively spliced transcript isoforms are commonly observed in higher eukaryotes. The expression levels of these isoforms are key for understanding normal functions in healthy tissues and the progression of disease states. However, accurate quantification of expression at the transcript level is limited with current RNA-seq technologies because of, for example, limited read length and the cost of deep sequencing.

**Results:**

A large number of tools have been developed to tackle this problem, and we performed a comprehensive evaluation of these tools using both experimental and simulated RNA-seq datasets. We found that recently developed alignment-free tools are both fast and accurate. The accuracy of all methods was mainly influenced by the complexity of gene structures and caution must be taken when interpreting quantification results for short transcripts. Using TP53 gene simulation, we discovered that both sequencing depth and the relative abundance of different isoforms affect quantification accuracy

**Conclusions:**

Our comprehensive evaluation helps data analysts to make informed choice when selecting computational tools for isoform quantification.

**Electronic supplementary material:**

The online version of this article (doi:10.1186/s12864-017-4002-1) contains supplementary material, which is available to authorized users.

## Background

Recent large genome-scale studies concluded that almost all human multi-exon genes could be spliced into multiple transcript isoforms [1]. There are 58,037 annotated human genes and 198,093 isoforms in Gencode v25 [2]. On average, there are 3.4 annotated transcripts per human gene and if only protein-coding genes are considered, the ratio increases to 7:1. However, the number of annotated transcripts does not fully represent the complexity of all alternative splicing events in cells. The available databases only annotate transcripts that are commonly observed. Novel transcripts are often discovered by RNA-seq, even in well-annotated organisms like human and mouse.

Isoform switching events are observed in various cellular processes, including tissue differentiation and transition from healthy to disease states [3–8]. Isoforms from the same gene can be involved in distinct processes or even play opposite roles. The p53 tumour suppressor gene also known as *Tumour Protein P53* (*TP53*) is well studied and has a central role in the regulation of DNA-damaged cells. *TP53* is frequently mutated in most human cancer types [9, 10]. However, not all *TP53* isoforms have the same role in tumour suppression. For instance, the roles of Δ133p53 and full-length p53β isoforms are opposite to each other. The Δ133p53 isoform inhibits apoptosis of tumour cells induced by the full-length p53β isoform [11, 12]. In such cases, it is essential to obtain accurate quantification of expression at the transcript level to understand the relative contribution of each isoform to a physiological state.

Our previous study [13] showed that a transcript-based approach led to a significant improvement in the accuracy of gene expression quantification over traditional union-exon based methods such as HTseq [14] and featureCounts [15]. Thus, transcript level quantification is recommended for all RNA-seq data analysis. Moreover, isoform quantification not only detects isoform-switching events that are masked by gene level analysis, but also improves gene level quantification accuracy by aggregating the transcript level quantification results [16, 17].

In recent years, RNA-seq has emerged as a powerful transcriptome profiling technology that allows in-depth analysis of alternative splicing [18]. In a typical RNA-seq assay, extracted RNAs are reverse transcribed and fragmented into cDNA libraries, which are sequenced by high throughput sequencers. Transcript isoforms coming from the same gene are highly similar in sequence and share a large percentage of overlapping regions. It is, therefore, a challenging task to identify the true origin of the short sequencing reads, given that reads from overlapping regions can come from any of the transcript isoforms.

A number of packages have been developed to quantify expression at the transcript level [19]. RSEM [20] implements iterations of EM (Expectation-Maximization) algorithms to assign reads to the isoforms from which they originate. eXpress [21] is a more recent tool that utilizes an online EM algorithm to improve the convergence speed of standard EM methods. TIGAR2 [22] utilizes Bayesian inference and aims to provide better accuracy for longer reads. Cufflinks [3] is a popular tool for novel transcript discovery and quantification. It attempts to explain the observed reads with a minimum number of isoforms. The strategy is similar to one iteration of the EM algorithm used in RSEM [20].

Most Recently, ultra-fast alignment-free methods, such as Sailfish [23], Salmon [24] and Kallisto [25], have been developed by exploiting the idea that precise alignments are not required to assign reads to their origins. Kallisto introduced a *de bruijn* graph to achieve efficient “pseudo-alignment” by checking the compatibility between short reads with transcripts. Sailfish was initially implemented using a k-mer approach, but was later improved to incorporate the same mapper from Salmon for “quasi-mapping”. Salmon implemented a two-phase inference procedure including both online and offline iterations of EM. Salmon is also a flexible tool that has two modes of quantification. It can either process sequence reads directly using its own mapper, i.e. RapMap [26], or it can take transcriptome-mapped BAM files as inputs. To distinguish these two running modes, the two modes are evaluated separately, with the former termed as “Salmon” and the latter termed as “Salmon_aln” in the following discussion.

In this paper, we performed a comprehensive evaluation of these tools using both experimental and simulated datasets, and investigated the impact of gene structural features on the accuracy of isoform quantification. Our evaluation focused on isoform quantification methods that aim to accurately quantify known transcripts. Thus, those methods that focus on novel transcript discovery, such as Stringtie [27], SLIDE [28] and iReckon [29], were excluded from this evaluation. After careful literature review, a total of seven tools were selected: Cufflinks, RSEM, TIGAR2, eXpress, Sailfish, Kallisto and Salmon. We used RSEM simulated datasets to measure the accuracy of methods, technical replicates of experimental data to test the robustness, and simulated transcripts from the *TP53* gene to illustrate the challenges of isoform quantification.

## Methods

### Datasets

The RNA-seq dataset for two technical replicates from Universal Human Reference RNA (UHRR-C1 and UHRR–C2) and two technical replicates from Human Brain Reference RNA (HBRR-C4 and HBRR-C6) were downloaded from Illumina’s BaseSpace. The four samples were prepared by a strand-specific protocol and deeply sequenced on a HiSeq 2500 platform, with about 80 million paired-end reads per sample. The RSEM package was used to simulate 50 million reads from the HBRR-C4 sample in the experimental dataset. The fraction of reads coming from “noise” (theta0) was set to 0.007 in the simulation.

### Workflow of quantification

**Fig. 1:**
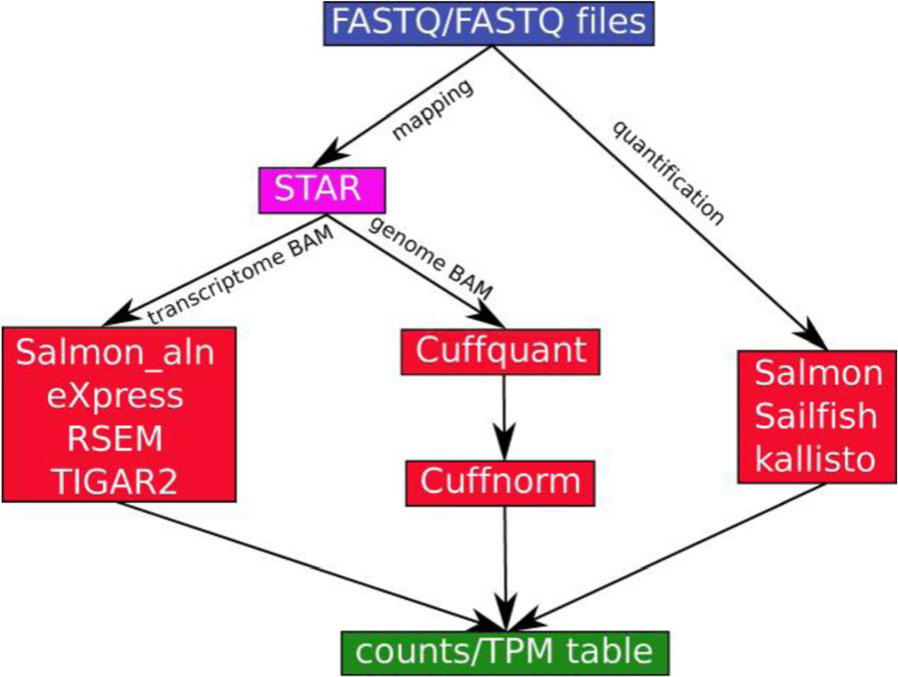
Workflow for transcript isoform quantification. Sequencing reads were either mapped by STAR aligner or directly fed into alignment-free methods, Salmon, Sailfish or Kallisto. The transcriptome BAM files were quantified by Salmon_aln, eXpress, RSEM or TIGAR2. The genome BAM files were quantified by Cuffquant and then Cuffnorm from the Cufflinks package. The results are summarized into counts and TPM tables for comparison

The transcript expression levels in both simulated and experimental datasets were quantified by the workflow depicted in Fig. 1. For each algorithm, detailed command line parameters are provided in the Additional file 1: Supplementary Methods. The initial input files for the workflow were sequence reads in FASTQ/FASTA format and the final output files were the summarized counts or TPM (Transcripts Per Million) tables. Some methods, including RSEM, TIGAR2, and eXpress, require transcriptome-mapping BAM files as input, while Cufflinks requires genome-mapping BAM files as input. To minimize the influence of mapping on quantification, we chose STAR [30] as the mapper, which has the capability to output both transcriptome-mapping and genome-mapping BAM files simultaneously. As we have demonstrated previously, the choice of a gene model has a dramatic impact on gene and isoform quantification [31, 32]. Built upon Ensembl [33] with improved coverage and accuracy, Gencode annotation has been used by the ENCODE consortium as well as many other projects (e.g., 1000 Genomes) as the reference gene set. We therefore chose the latest Gencode annotation (v25 at the time of writing) for this evaluation.

### Evaluation protocol for RSEM simulated data

Estimated counts and TPM values were compared against simulated “ground truth” values. Pearson correlation coefficient (R^2^) and MARDS (Mean Absolute Relative Differences) were quantified and compared across methods. Lowly expressed transcripts are “noisy” and all estimated counts below 5 were considered not expressed and were set to zero. Next, transcripts with 0 estimated counts for all methods were removed, to prevent “inflation” of MARDS and R^2^ calculation. Accordingly, 92,139 transcripts out of 198,093 annotated transcripts in Gencode v25 survived this filtering.

Raw counts and TPM values were log_2_ transformed for R^2^ calculation. To avoid arithmetic error and large negative values in log_2_ transformation, a pseudo-count of 5 was added or 0.1 TPM were added to all transcripts in the counts or TPM tables prior to transformation. The purpose of adding a pseudocount is to avoid computational error or a very large negative number in log2 transform when the expression is zero or very small. We tried 0.01, 0.1, 0.5 and 1 as pseudo-counts, and the conclusion in this paper does not change.

For each transcript, let ***i*** be the simulated count and ***j*** be the estimated count. Absolute relative difference (ARD) was calculated as:

$$ ARD=\kern0.5em \left\{\begin{array}{c}\hfill \frac{\left|i-j\right|}{i+j}\ \left( if\ i+j\ne 0\right)\hfill \\ {}\hfill 0\ \left( if\ i=j=0\right)\hfill \end{array}\right. $$

Then, MARDS were calculated as the arithmetic mean of ARD.

For false positive rate calculation, the counts table was filtered to contain only non-expressed transcripts. There were 99,202 transcripts with 0 simulated counts. A false positive was determined when the simulated count is 0 but the estimated count is above 5. For TPM tables, 0.1 was used as the cut-off.

### Evaluation protocol for experimental data

TPM values were estimated for each experimental dataset. A pseudo-count of 0.1 was added to each transcript before log_2_ transformation. No filtering was applied when calculating correlation between technical replicates. The correlation between HBRR-C4 and HBRR-C6 was calculated across methods, so was the correlation between UHRR-C1 and UHRR-C2.

Next we compared the pairwise correlation across methods by using the same sample, HBRR-C4. TPM values estimated by all methods were summed up for each transcript during analysis. Transcripts with a sum less than 0.8 were considered “noisy” and thus excluded from analysis. Since there are eight methods, the cutoff 0.8 amounts to 0.1 per sample, which is consistent with the pseudocount added in log2 transformation.

### *TP53* transcripts simulation

The Bioconductor package, *polyester* [34], was used to simulate paired-end strand-specific reads coming from six isoforms (α, β, γ and Δ133α, Δ133β, Δ133γ) of the *TP53* gene with default options, and 100 simulated reads were generated for each transcript at the base line. To evaluate the impact of read depth, we then increased the number of reads for all six transcripts 10 and 100 fold. To evaluate the impact of relative abundance on the accuracy of quantification, we increased only the number of reads for FLα (full-length transcript α) 10 and 100 fold, while the number of reads for the other five transcripts were kept at 100. Each condition was simulated five times and MARDS were calculated from the mean of the five replicates. All reads were randomized before mapping and quantification, and fake quality scores were added to all simulated reads. Because the total number of reads in each simulation was small, the online-phase “Burn-in” was turned off in Salmon and Salmon_aln by setting “--*numPreAuxModelSamples 0*”.

## Results

Fifty million paired-end reads were generated by the RSEM RNA-seq simulator based upon the read distribution statistics taken from experimental sample HBRR-C4. Table 1 summarizes the features and running metrics for all the eight methods (seven packages plus Salmon_aln). TIGAR2 required a lot more memory and more time than the other methods, while the alignment-free methods (Salmon, Sailfish and Kallisto) were clearly the fastest. All methods use iterations of the EM algorithm for quantification. RSEM, Kallisto, eXpress and Cufflinks use the Maximum Likelihood objective (ML), while TIGAR2 uses the Variational Bayes objective (VB). Salmon and Sailfish allow users to choose which objective to use. In this evaluation, we used the default ML objective.

**Table 1:** Run time metrics of each method on 50 million paired-end reads of length 76 bp in an high performance computing cluster

	Memory (Gb)	Run time (min)	Algorithm	Multi-thread
Cufflinks	3.5	117	ML	Yes
RSEM	5.6	154	ML	Yes
eXpress	0.55	30	ML	No
TIGAR2	**28.3**	**1045**	VB	Yes
kallisto	3.8	7	ML	Yes
Salmon	6.6	6	VB/ML	Yes
Salmon_aln	3	7	VB/ML	Yes
Sailfish	6.3	5	VB/ML	Yes

For methods that support multi-threading, eight threads were used. For alignment-free methods (Kallisto, Salmon and Sailfish), a mapping step was included. The best performer in each category is underlined and the worst performer is in bold

*ML* Maximum Likelihood, *VB* Variational Bayes

### Comparisons of isoform quantification accuracy across methods

**Fig. 2:**
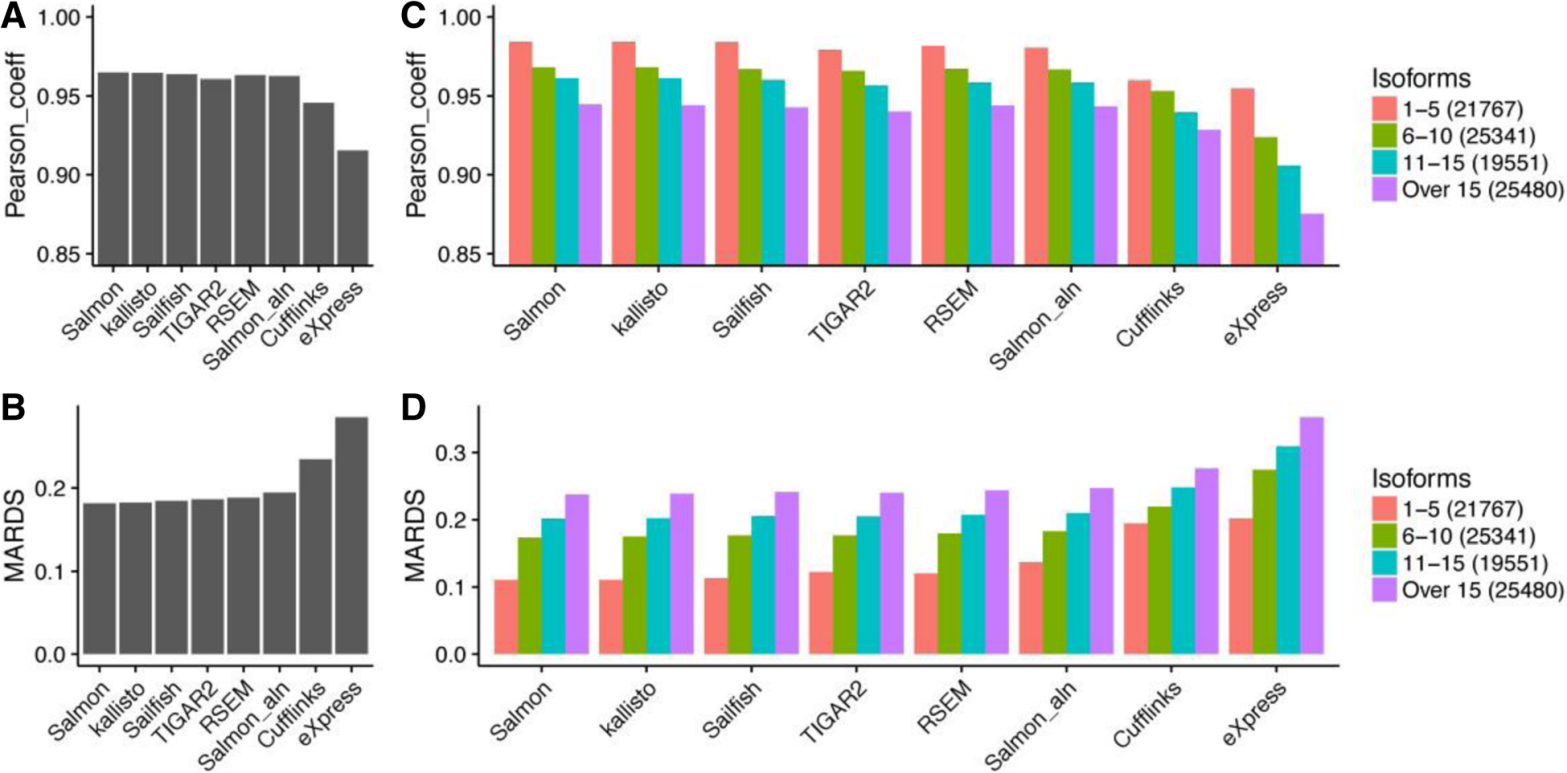
Comparisons of the overall performance among different methods and the impact of the number of transcripts on the accuracy of isoform quantification. **a** Pearson correlation coefficient. **b** mean absolute relative differences and **c-d**) The above metrics were broken into separate groups according to the number of annotated transcript isoforms for each gene. The number of transcripts in each group is shown in figure legends. The accuracy metrics were calculated by comparing the estimated counts with the “ground truths” in simulated dataset

We first filtered out lowly expressed transcripts and log_2_ transformed the counts and TPM tables as described in the Methods Section. Then, R^2^ and MARDS were calculated as accuracy measurements for expressed transcripts using estimated read counts (Fig. 2a and b) and TPM values (Additional file 1: Figure S1A and B). R^2^ is a good metric for global agreements between two sets. It is robust against outliers after log_2_-transformation, but does not give a good estimate if there is strong linear bias. MARDS, on the other hand, is a local measurement for relative errors. It can detect global biases, but is not robust against outliers. By combining the two metrics, we obtained a comprehensive view of the accuracy measurement of the eight methods from the seven chosen tools. We also calculated Spearman correlation coefficient and RMSD (Root Mean Squared Distance) described by Teng et al. [35] (Additional file 1: Figure S2A and B), however, we did not observe any additional benefits.

Figure 2a and b show strong agreements between R^2^ and MARDS. In general, the higher the R^2^, the smaller the corresponding MARDS. Overall, all methods had a good performance by achieving R^2^ over 0.91 and MARDS less than 0.3. Cufflinks and eXpress, showed worse scores in both categories, and performed worse than the other methods in this simulation. The accuracy difference was small for the other six methods, achieving R^2^ over 0.95 and MARDS less than 0.2. The same conclusions can be drawn using either counts or TPM values.

### The impact of gene complexity on the accuracy of isoform quantification

Next, we investigated what features impact the accuracy of transcript quantification. One such feature is the structural complexity of a gene. If a gene has a complex structure, with a large number of highly similar transcript isoforms, it can be difficult for algorithms to correctly assign reads to their true origins. To quantify this effect, we divided the transcripts evenly into four separate groups according to the number of isoforms of their corresponding genes (1–5, 6–10, 11–15 and above 15). In general, a gene becomes more complex as the number of annotated isoforms increases. We measured the R^2^ and MARDS for each group (Fig. 2c and d). There was a solid trend that the quantification accuracy decreased as the number of isoforms increased. Cufflinks had the smallest reduction in R^2^ and MARDS measurements (R^2^ decreased by 0.013 and MARDS increased by 0.057). eXpress was most sensitive to gene structures, with R^2^ decreased by 0.056 and MARDS increased by 0. The number of annotated exons in a gene is another good descriptor of gene structure complexity. We divided the transcripts similarly according to the number of exons in the transcripts (1–5, 6–10, 11–20 and above 20) and drew a similar conclusion that the quantification accuracy decreases as the number of exons increases (Additional file 1: Figure S3).

### Disagreement in effective transcript length for short transcripts

**Fig. 3:**
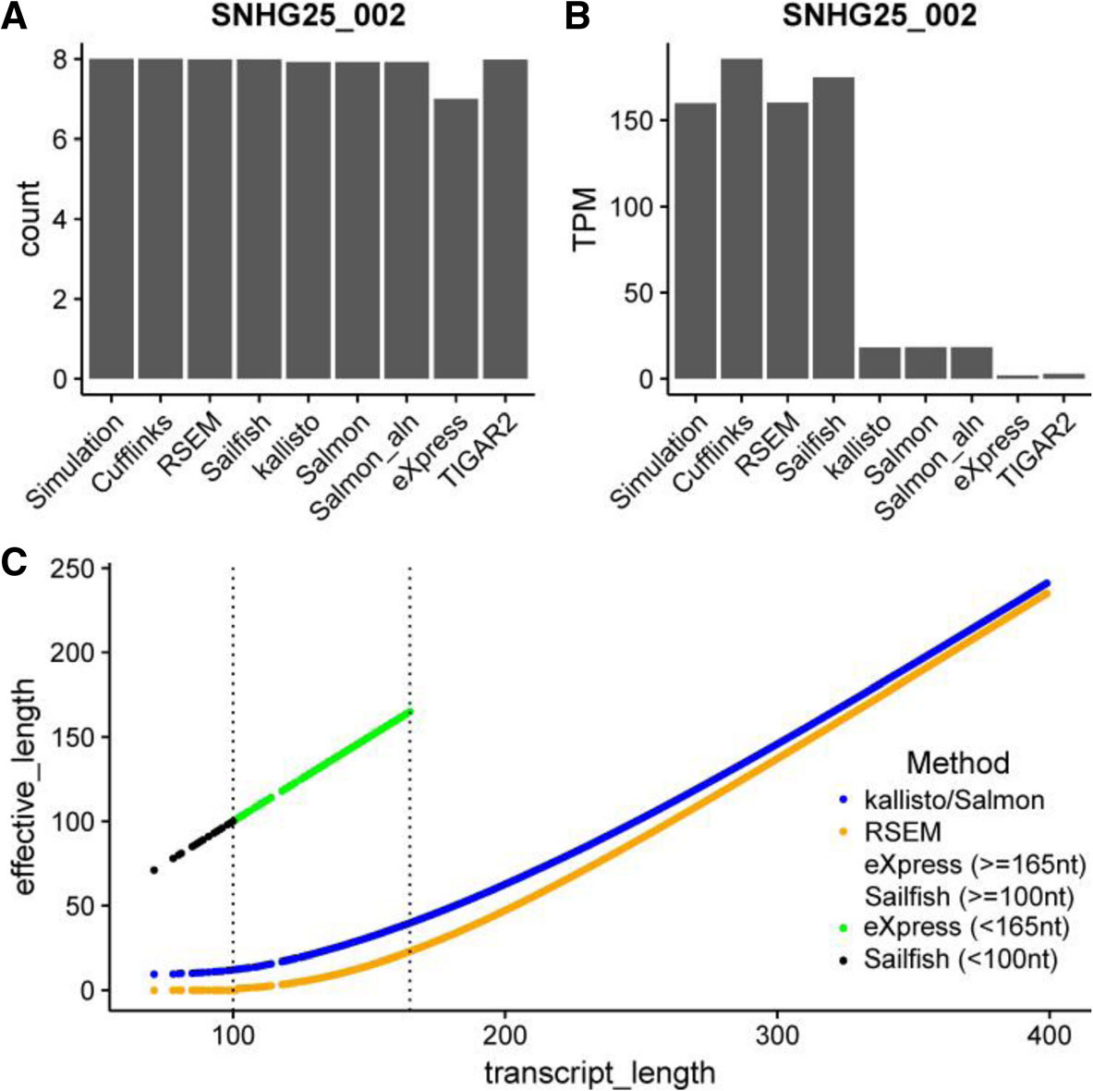
Inconsistency in effective length calculation among methods for short transcripts. **a** Read counts were estimated correctly for the transcript SNHG25–002. **b** Methods showed disagreement in estimating TPM values for the same transcript. **c** The relationship between the effective transcript length estimated by each method and the corresponding transcript length. Only transcripts with length less than 400 nt are shown. Note the transcript length at x-axis is the total number of nucleotides of the transcript in the Gencode Release v25

TPM values, calculated using the estimated read counts normalized against effective transcript length and total number of reads, are a good measurement of transcript expression levels in a sample and are recommended to replace FPKM values (Fragments Per Kilobase Per Million) [36, 37]. In principle, the same conclusions should be drawn regardless of the choice of counts or TPM values. However, there are some cases where the counts are estimated correctly but the corresponding TPM values are not. Transcript SNGH25–002 is a case in point. Eight reads were simulated for this transcript, and almost all methods estimate the count accurately. However, the TPM values range from 1.98 to 185.55, a difference of two orders of magnitude (Fig. 3a and b). Other examples giving similar results include transcripts RNY3–201 and Y_RNA.490–201 (Additional file 1: Figure S4A, B, C and D). The transcripts SNGH25–002 and RNY3–201 were found at top when comparing the estimated TPMs across methods and with ground truths, and thus were chosen to demonstrate the issue with TPM estimation of short transcripts.

After further investigation, we noted that all three transcripts (SNGH25–002, RNY3–201 and Y_RNA.490–201,) have a transcript length around 100 nt and the heterogeneous TPM values result from disagreement in calculations of the effective transcript length for short transcripts. An effective transcript length is determined by the transcript length and the empirical fragment length distribution in a sample [21, 24]. It was introduced to accommodate the limited range of cDNA fragment sizes that can be sampled near the two ends of a transcript. For large transcripts, the estimated effective lengths were similar among different methods. However, for transcripts with length close to or less than the average fragment length, there was no consensus model to estimate the effective length. By plotting the transcript length against estimated effective length, Fig. 3c shows three different models that are commonly applied in this situation and that produce vastly different estimates when the transcript length is short. All methods gave similar estimates for transcripts over 300 nt. For transcripts of less than 300 nt, Kallisto and Salmon follow the same model, while RSEM, Sailfish and eXpress use a different model. While RSEM makes corrections for all transcript lengths, eXpress uses the actual transcript length as their effective length for transcripts less than 165 nt. For Sailfish, the cut-off is lower at about 100 nt. Each model has its own merits and there is no “correct answer”. All transcripts with length shorter than 200 nt were extracted, and their corresponding accuracy metrics (see Additional file 1: Figure S4E and F) were calculated using counts and TPM values, respectively. Apparently, the isoform quantification results for short transcript have much lower Pearson correlation and larger MARDS, with TPM values showing more heterogeneity. Transcripts shorter than the fragment lengths are filtered out during library preparation. Thus, RNA-seq is not a good method for the measuring the expression of very short transcripts.

### Comparisons of robustness and consistency of isoform quantification across methods

**Fig. 4:**
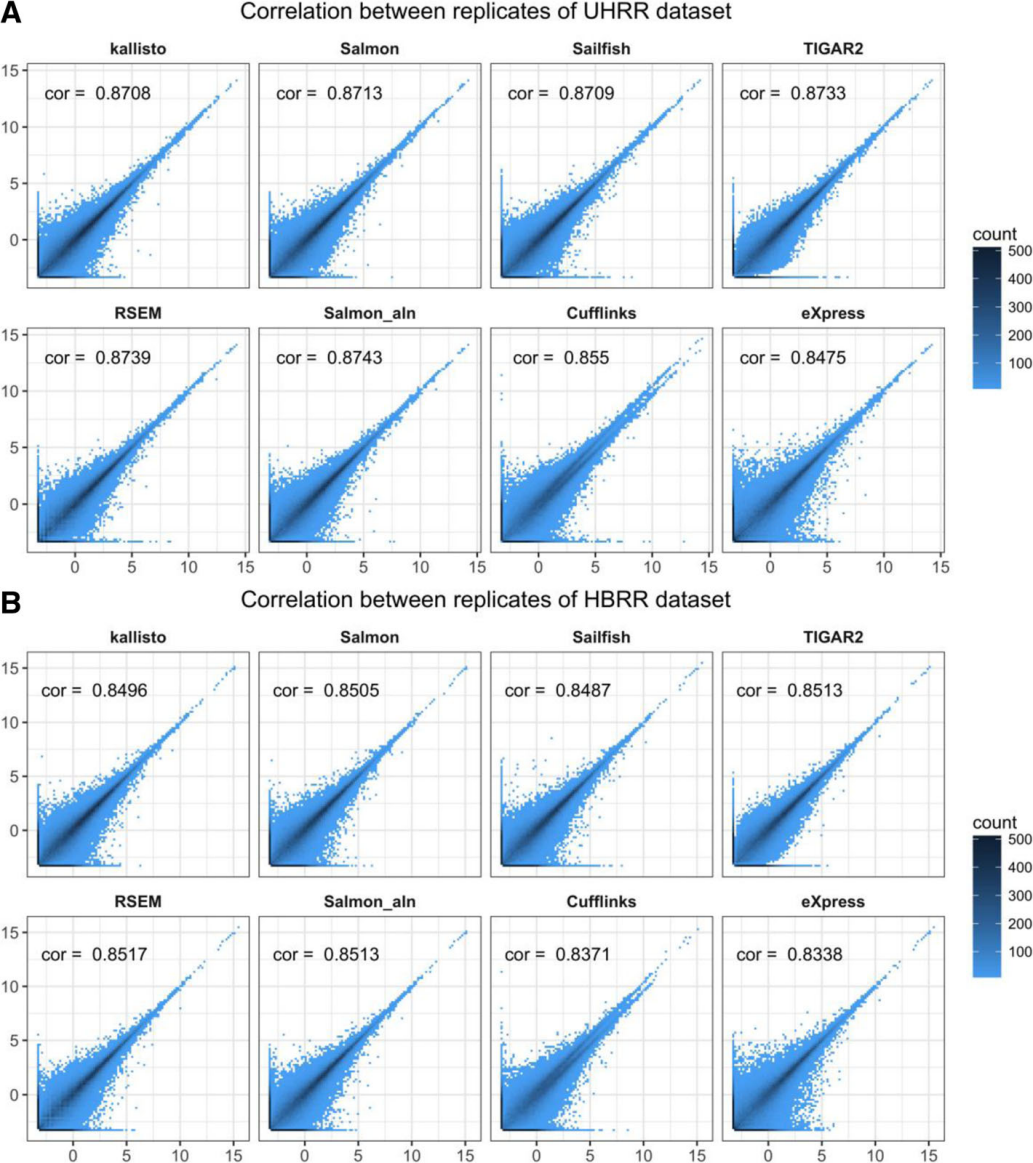
Correlation of estimated TPM values for all transcripts between technical replicates of experimental datasets. **a** UHRR-C1 (x-axis) and UHRR-C2 (y-axis). **b** HBRR-C4 (x-axis) and HBRR-C6 (y-axis). The *R* ^*2*^ value is shown in each figure. Note, x and y-axes represent log_2_ transformed estimated transcript TPM values

Another important metric is the consistency between technique replicates. Ideally, quantification results should be close for technical replicates from the same RNA sample. HBRR and UHRR experimental datasets are selected for this purpose. The scatter density plots for estimated TPM values between UHRR-C1 and UHRR-C2, and those between HBRR-C4 and HBRR-C6 are shown in Fig. 4a and b, respectively. RNA sequencing is intrinsically a random process, and estimated TPMs are not exactly the same between replicates and some variations are expected, especially for lowly expressed genes or transcripts. The dark colour arrayed along the diagonal line indicates good correlation between replicates for all methods. While there is a certain amount of noise in estimating lowly expressed transcripts, highly expressed transcripts show strong concordance between replicates. The high R^2^ values validate the robustness of each computational method.

**Fig. 5:**
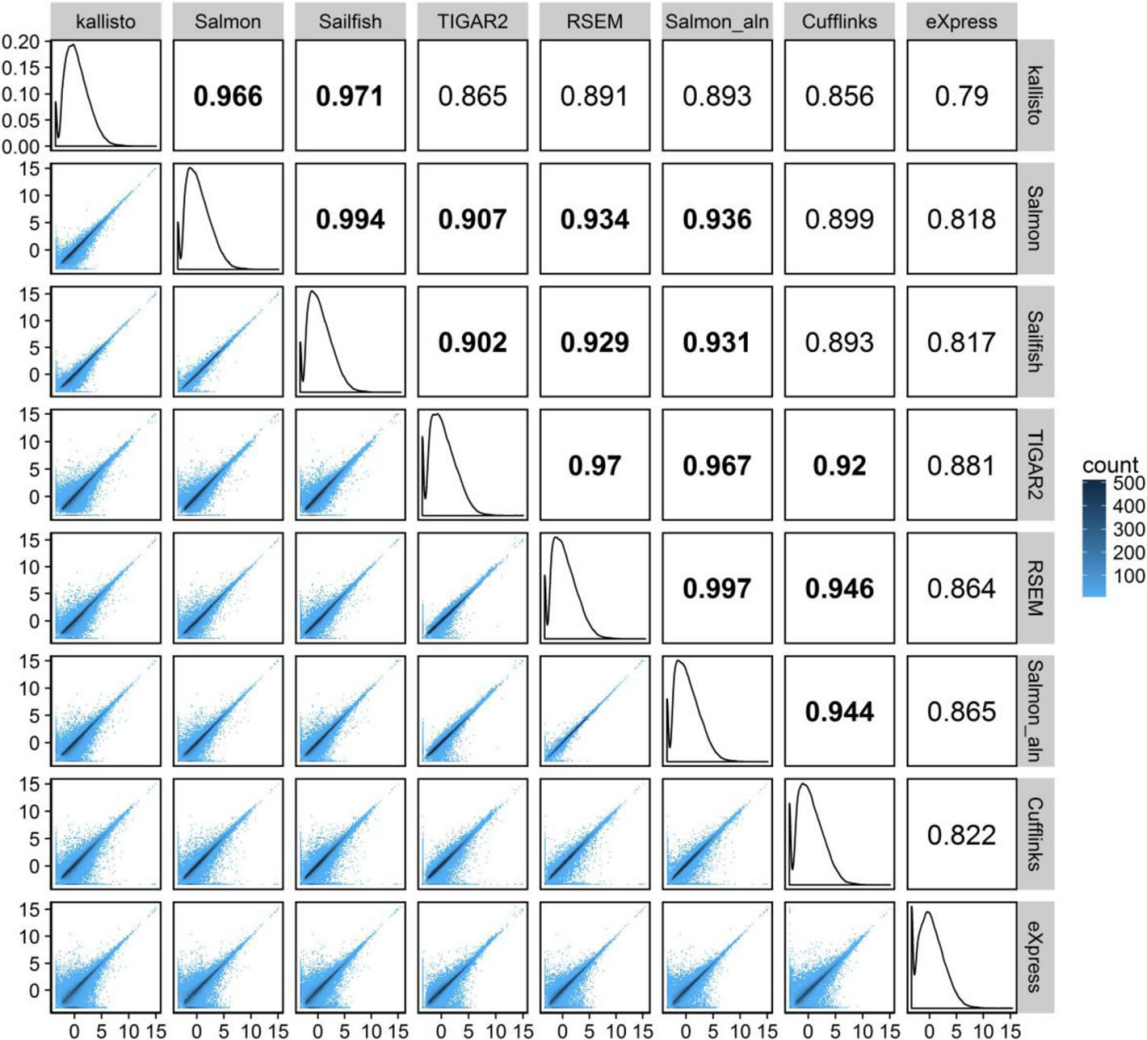
Pairwise correlation of estimated TPM values for all transcripts between methods for the HBRR-C4 sample. The distribution of transcripts’ TPMs from each method was plotted on the diagonal panels. Pairwise density plots and *R* ^*2*^ values are shown in the lower and upper triangular panels, respectively. *R* ^*2*^ values over 0.9 are in *bold*. Methods are grouped using hierarchical clustering

*R* ^*2*^ values and density plots of estimated TPMs between each pair of methods for the sample HBRR-C4 are summarized in Fig. 5. Overall, methods showed strong concordance with one another, especially for highly expressed transcripts. eXpress produced the most disparate results compared with the other methods. The three alignment-free methods, Salmon, Sailfish and Kallisto, cluster tightly together with *R* ^*2*^ > 0.96. Salmon_aln and Salmon use the same quantification algorithms but different aligners, and the strong agreement between their estimated counts (*R* ^*2*^ = 0.936) indicates the choice of mapping methods has only a mild impact on transcript quantification. Interestingly, Salmon_aln showed stronger agreement with RSEM than with Salmon (*R* ^*2*^ = 0.997). This is because Salmon_aln and RSEM both use STAR as aligner and the EM algorithms implemented in these two packages give very close estimations.

### Impact of mapping algorithm on the accuracy of isoform quantification

For fair comparisons, we chose STAR as the only aligner for alignment-dependent quantification methods. However, the three alignment-free methods use their own mappers for pseudo-alignment or quasi-mapping, which gives an opportunity to explore the impact of different mapping methods. In particular, Salmon gives the flexibility of choosing either its internal RapMap mapper or external aligners, while keeping the quantification step similar. As shown in Fig. 5 for the sample HBRR-C4, there is strong concordance among quantification results from RSEM, Salmon, Salmon_aln, Kallisto and Sailfish (*R* ^2^ > 0.89), indicating that the impact of mappers on isoform quantification is small.

**Fig. 6:**
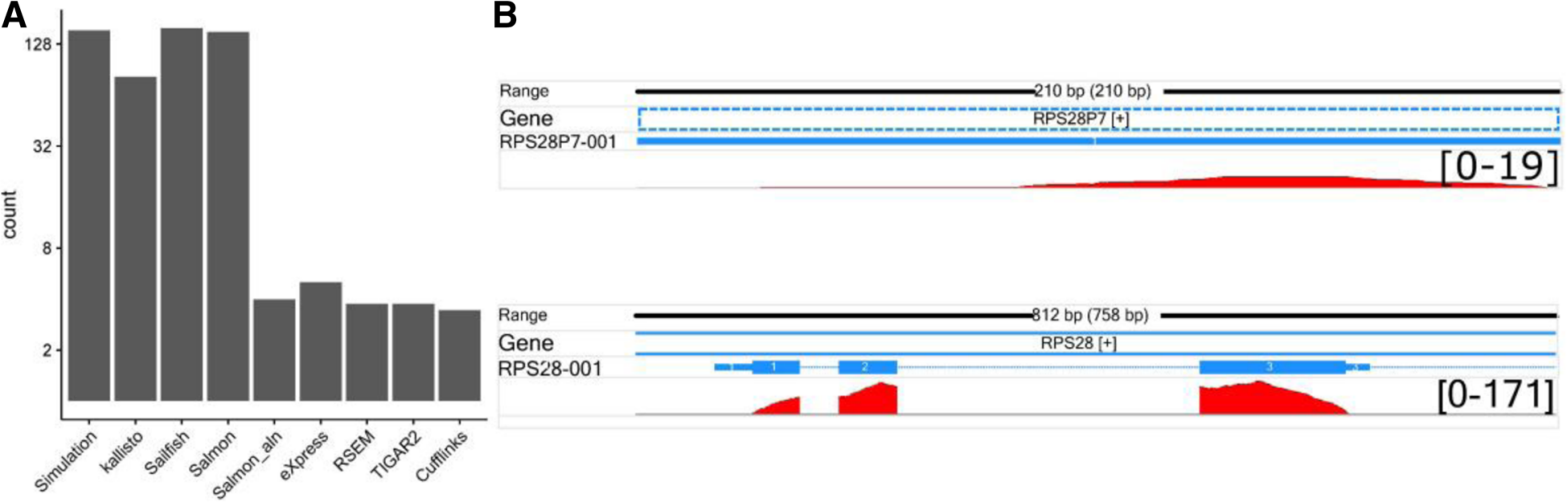
Significant difference in estimated read counts for transcript RPS28P7–001 resulting from STAR aligner. A total of 154 reads for RPS28P7–001 were simulated. **a** The estimated read counts from all eight methods are shown, and they are severely underestimated by the methods using STAR aligner. **b** The read coverage profiles (coloured in red) in RPS28P7–001 and RPS28–001. The peak paired-end read counts (both ends counted) are shown in brackets. Only a small fraction of reads were mapped back to the *RPS28P7* region while the majority of reads were incorrectly mapped to the *RPS28* gene

By looking deeper into the RSEM simulation dataset, we found a few cases in which the choice of mappers did make a big difference. For instance, reads from transcript RPS28P7–001 were vastly underestimated in all methods using STAR aligner (Fig. 6a). We extracted all reads coming from RPS28P7–001 and used STAR to map them to the human genome. Surprisingly, the resulting BAM file indicated that the majority of the reads were uniquely mapped to the gene *RPS28* instead of *RPS28P7* (Fig. 6b). *RPS28P7* is a pseudogene of *RPS28*. Although the two transcripts share the same partial sequence, RPS28–001 is a spliced isoform, while RPS28P7–001 is not. STAR, by default, adds bonus scores to spliced alignments to discourage mapping of reads to pseudogenes. This strategy does well in most cases in experimental datasets, because pseudogenes tend to have no or low expression compared to their canonical counterparts. However, in our evaluation, it results in undesirable mapping, and accordingly, dramatically underestimates the expression level of the transcript RPS28P7–001.

### Impact of sequencing depth and relative abundance on the accuracy of isoform quantification

**Fig. 7:**
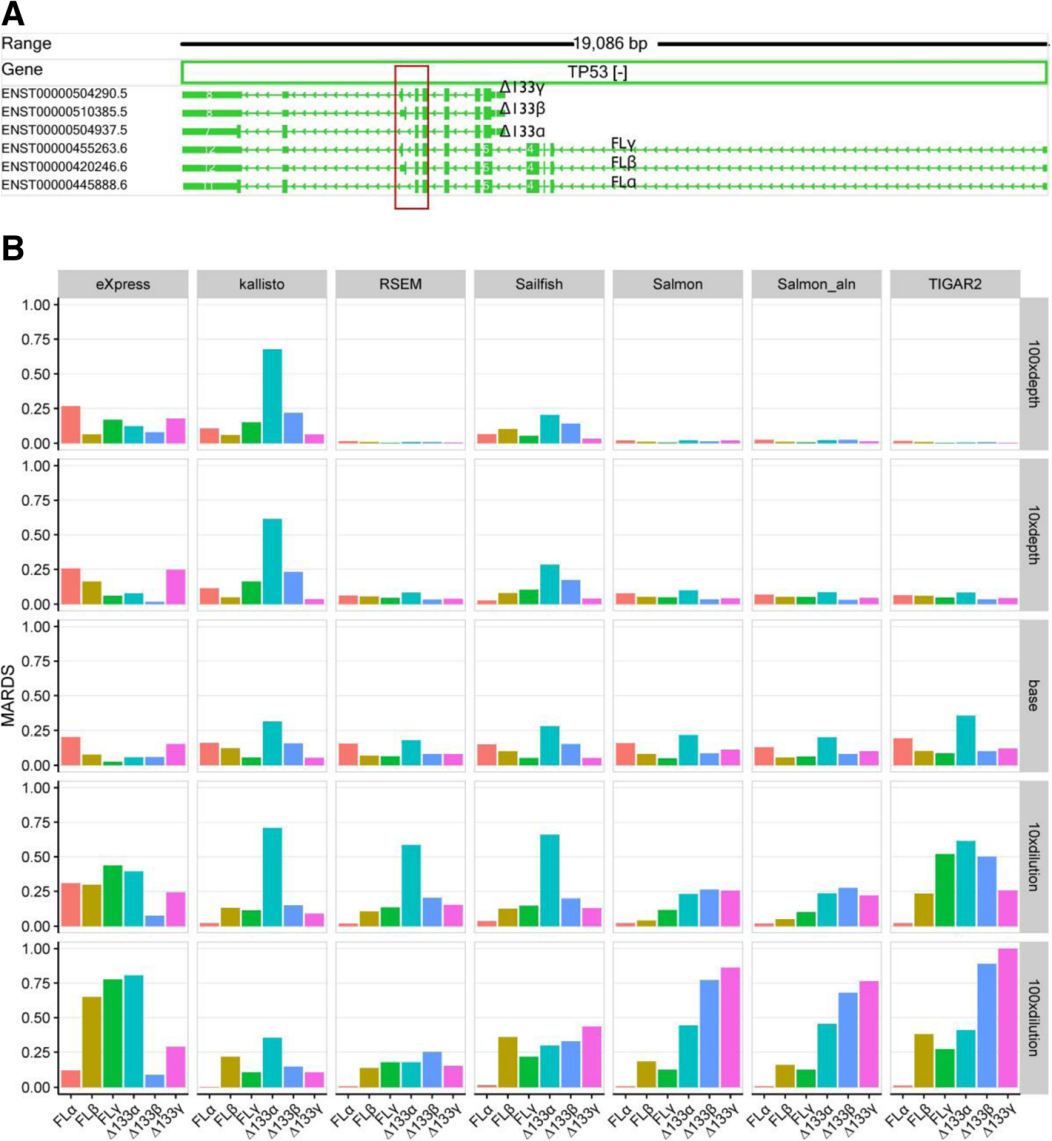
The impact of sequencing depth and relative abundance on the accuracy of isoform quantification. **a** Structures of six canonical transcripts of the *TP53* gene, and their corresponding identifier in GENCODE v25. **b** The accuracy of isoform quantification with each of the seven methods under each simulation condition. MARDS was calculated using known and estimated read counts

The *TP53* gene encodes several different transcripts and plays important roles in multiple cancer types. As illustrated in Fig. 7a, *TP53* is encoded on the minus strand of human chromosome 17. Alternative promoter usage results in either a full-length (FL) or a truncated transcript (Δ133). Alternative exon usage in the middle box region results in α, β and γ variants. Canonical splicing events of this gene give rise to six transcript isoforms (FL α, β, γ and Δ133 α, β, γ) [9, 10]. Non-canonical transcript isoforms in Gencode v25 annotation are not included in this paper.

To further investigate the impact of sequencing depth and relative abundance on the accuracy of isoform quantification, we chose *TP53* and performed a controlled simulation as detailed in the Methods Section. Figure 7b shows the MARDS metrics for each transcript from each of the seven methods at each simulated condition. Cufflinks crashed when quantifying this simulation and was thus excluded from this comparison. The results corresponding to the base line are shown in the third row of Fig. 7b.

As the sequencing depth increases (the top three rows in Fig. 7b), the accuracy improved for RSEM, Salmon, Salmon_aln and TIGAR2, but not for Sailfish and the accuracy actually decreased for eXpress and Kallisto. After discussions with the developer of Salmon and Sailfish, we think this is caused by how reads are modelled in their EM algorithms. While Kallisto and Sailfish consider only the effective transcript length when assigning reads that are compatible to more than one isoforms, RSEM and Salmon also consider the current estimated abundance of each isoform as the prior. The former model performed well in most cases, as evident in the RSEM simulated dataset in the previous sections, but in this particular case, it had relatively poor performance when the sequencing depth was high.

Genes tend to have one isoform highly expressed with other isoforms expressed at low levels. For instance, the sum of TPM values of the most abundant isoform in each gene takes up 76.6% of the total TPM values of all transcripts in the HBRR-C4 sample. The detailed distribution of the ratios of the TPM values between the most abundant isoforms and their corresponding genes in HBRR-C4 are shown in Additional file 1: Figure S5. The impact of the relative abundance of different transcripts on isoform quantification is less well explored in previous studies. According to our evaluation, the accuracy of FLα improves consistently as the relative abundance of FLα increases (see the bottom three rows of Fig. 7b), while the accuracy of the other five transcripts decreases. Kallisto, RSEM and Sailfish were the best performers with MARDS <0.5 (bottom row) in the most imbalanced situation. As imbalanced expression is commonly observed in isoform expression profiles, the large error rates in the bottom row suggest challenges remain for accurate quantification of minor transcript isoforms.

## Discussion

### Caution on quantification of short transcript and lowly expressed transcripts

All methods used in this study give highly reproducible results when technical replicates are used, especially for transcripts with high expression levels. In RSEM simulation data, transcripts with estimated counts less than 100 have relatively high variance and the results should be used with caution. Transcripts shorter than the fragment lengths are excluded during library preparation. As a result, caution must be taken when interpreting quantification results for short transcripts. The RSEM simulation was taken from an experimental dataset and only 138 transcripts of less than 200 nt were expressed in the simulation, with only 37 having read counts over five. In our simulation, the number of short transcripts included was too small to significantly impact the overall conclusion in the accuracy measurements.

### Impact of gene structure on the accuracy of quantification

Using an RSEM simulated dataset, we reached different conclusions from Kanitz et al. on the impact of gene structures on quantification accuracies [17]. Specifically, in their simulated dataset, when the number of isoforms or exons increased, there was no clear trend of decreasing accuracies. We think the difference in conclusions is due to different simulation strategies. In their *Flux-simulator* [38] simulation, the transcripts to be expressed were picked by random, many of which were short transcripts. They also explained that 60% of the transcripts with only one exon are short transcripts. Here, we showed that there is no consensus for estimation of these short transcripts and the results are inaccurate and difficult to interpret. We think the existence of a large number of short transcripts could obscure the trend that was revealed in their study. In our simulation, expression was determined from the HBRR-C4 sample. As mentioned above, only 138 short transcripts were included in the simulated dataset. In this sense, our conclusion holds true for the practical analysis of real experimental RNA-seq datasets.

### Impact of sequencing depth and relative abundance on quantification

We discovered both sequencing depth and relative abundances have strong impacts on quantification accuracy. Surprisingly, not all methods perform better when the absolute abundances increase. The abundance range explored here was close to true biological conditions, with around 10^2^ *TP53* reads in the HBRR datasets and 10^4^ *TP53* reads in the UHRR datasets. Isoform expression levels for most transcripts are also imbalanced in cells, with a few transcripts dominating the expression. Our simulation demonstrates that even with reasonable absolute abundances, all current methods have difficulty in accurately quantifying the expression levels of those isoforms whose relative abundancy is low.

## Conclusion

After a comprehensive evaluation of seven packages for isoform quantification, we found that alignment-free methods, such as Salmon, Sailfish and Kallisto, require less computational time while achieving similar or better accuracies compared with other methods. Cufflinks and eXpress, two alignment-dependent algorithms in our evaluation, have inferior accuracy performance with an RSEM simulated dataset. TIGAR2 has overall good performance, but the run time and memory requirements render the tool less popular for practical use. Considering both the accuracy and computational resources needed, Salmon-aln and RSEM are the two best performers among the alignment-dependent tools.

## Additional file

Additional file 1: Supplementary Method. Detailed command line parameters. **Figure S1.** Comparisons of the overall performance among different methods using TPM measure. **Figure S2.** Comparisons of the overall performance among different methods using counts measure. **Figure S3.** The impact of the number of exons on the accuracy of isoform quantification. **Figure S4.** Inaccuracy of isoform quantification for short transcripts in RSEM simulated dataset. **Figure S5.** The distribution of the ratios of TPM values between the most abundant isoforms and their corresponding genes in the HBRR-C4 sample. (PDF 663 kb)
