# Supplementary material for: Evaluation and comparison of computational tools for RNA-seq isoform quantification
Source: BMC Genomics. 2017 Aug 7;18:583. doi: 10.1186/s12864-017-4002-1 (PMC5547501; doi:10.1186/s12864-017-4002-1)
Supplement: Additional file 1: — Supplementary Method. Detailed command line parameters. Figure S1. Comparisons of the overall performance among different methods using TPM measure. Figure S2. Comparisons of the overall performance among different methods using counts measure. Figure S3. The impact of the number of exons on the accuracy of isoform quantification. Figure S4. Inaccuracy of isoform quantification for short transcripts in RSEM simulated dataset. Figure S5. The distribution of the ratios of TPM values between the most abundant isoforms and their corresponding genes in the HBRR-C4 sample. (PDF 663 kb) [file 12864_2017_4002_MOESM1_ESM.pdf]

Evaluation and comparison of computational tools for RNA-seq isoform quantification

Chi Zhang, Baohong Zhang, Lih-Ling Lin, Shanrong Zhao

Supplementary Method

In our study, STAR version 2.5.2a was chosen as the mapper for alignment dependent quantification methods. It was specified to output both genome mapping and transcriptome mapping BAM files. The following parameters are specified:

–alignEndsType EndToEnd --quantMode TranscriptomeSAM --outSAMtype BAM SortedByCoordinate --alignSJDBoverhangMin 1 --outFilterMismatchNoverLmax 0.05 --outFilterScoreMinOverLread 0.90 --outFilterMatchNminOverLread 0.90 --alignIntronMax 1000000

Cufflinks version 2.2.1 was used to quantify reads from genome mapping BAM files with strand information using existing annotation. First, Cuffquant was used to quantify only annotated transcripts and output ".cxb" files with option "–multi-read-correct". Then, Cuffnorm was used to output normalized counts per million (CPM) values by taking ".cxb" files as input with default options.

Kallisto, Salmon and Sailfish were used to directly quantify isoform expressions from sequencing reads with strand information. Kallisto version 0.43.0 was used with default options; Salmon version 0.8.0 was used with option "--incompatPrior 0"; and Sailfish version 0.10.0 was used with option "--enforceLibCompat". The parameters were specified to exclude mapping of reads that are incompatible with the library strand information.

RSEM, eXpress, Salmon_aln and TIGAR2 were used to quantify reads from transcriptome mapping BAM files. Strand information was accepted except for TIGAR2. For eXpress, the BAM files were resorted by transcript names using samtools 1.3.1 [34]. RSEM version 1.2.31 was used with default options; eXpress version 1.5.1 was used with option "--no-bias-correct"; TIGAR2 version 2.1 was used with option "--alpha_zero 0.1"; and Salmon_aln was used with option "--incompatPrior 0".

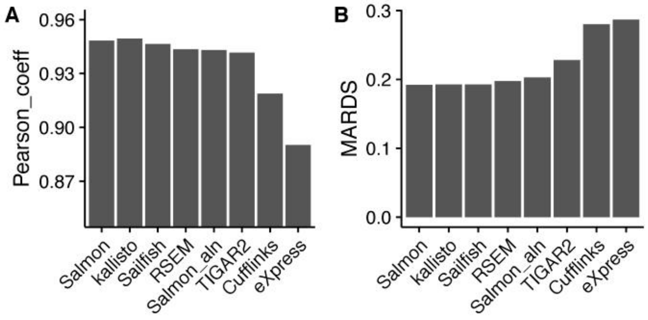

Figure S1. Comparisons of the overall performance among different methods using TPM measure. **A**) Pearson correlation coefficient; **B**) mean absolute relative differences. The metrics were calculated by comparing the estimated TPM values with the "ground truths" in simulated dataset.

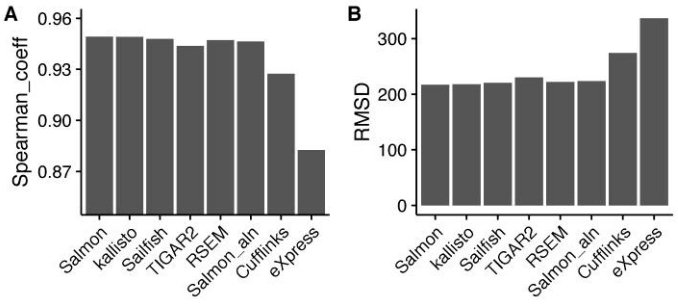

Figure S2. Comparisons of the overall performance among different methods using counts measure. **A**) Spearman correlation coefficient; **B**) RMSD (Root Mean Squared Distance). The metrics were calculated by comparing the estimated counts values with the "ground truths" in simulated dataset.

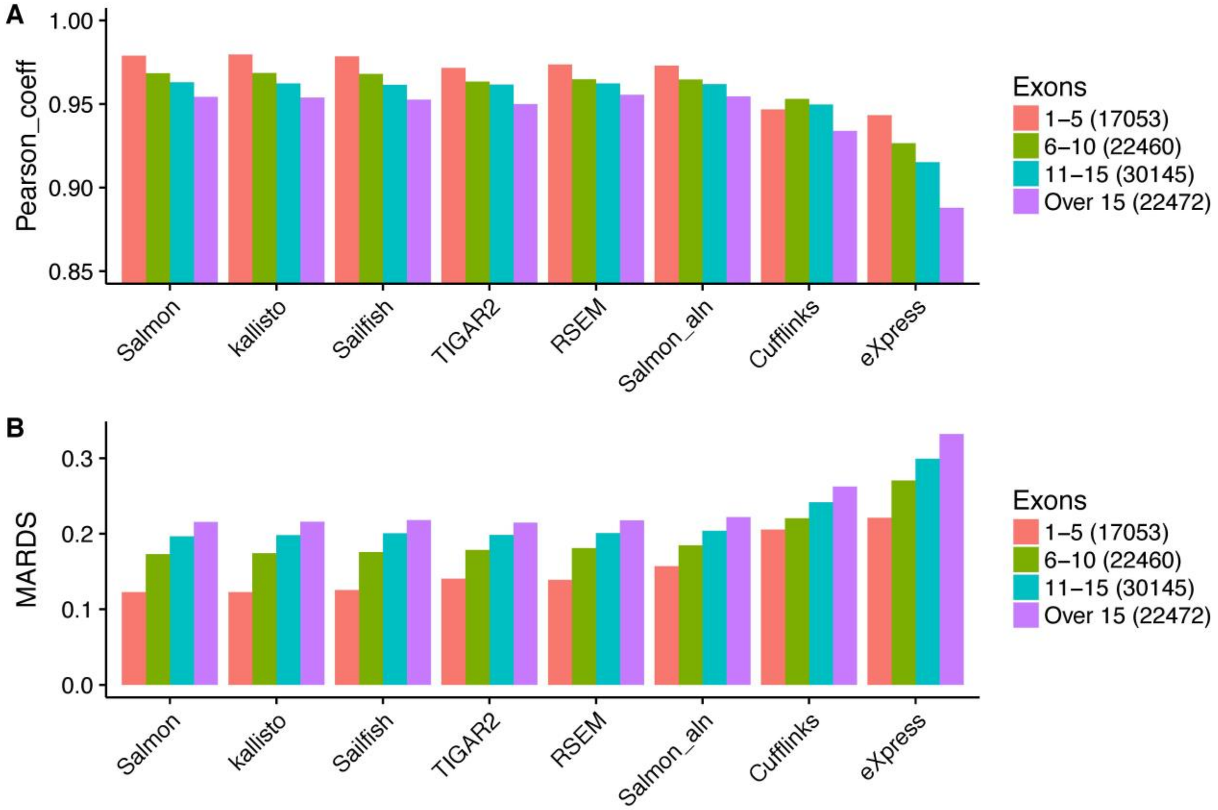

Figure S3. The impact of the number of exons on the accuracy of isoform quantification. **A**) Pearson correlation coefficient. **B**) mean absolute relative differences. All isoforms were broken into separate groups according to the number of annotated exons for each gene. The metrics were calculated by comparing the estimated TPM values with the "ground truths" in simulated dataset.

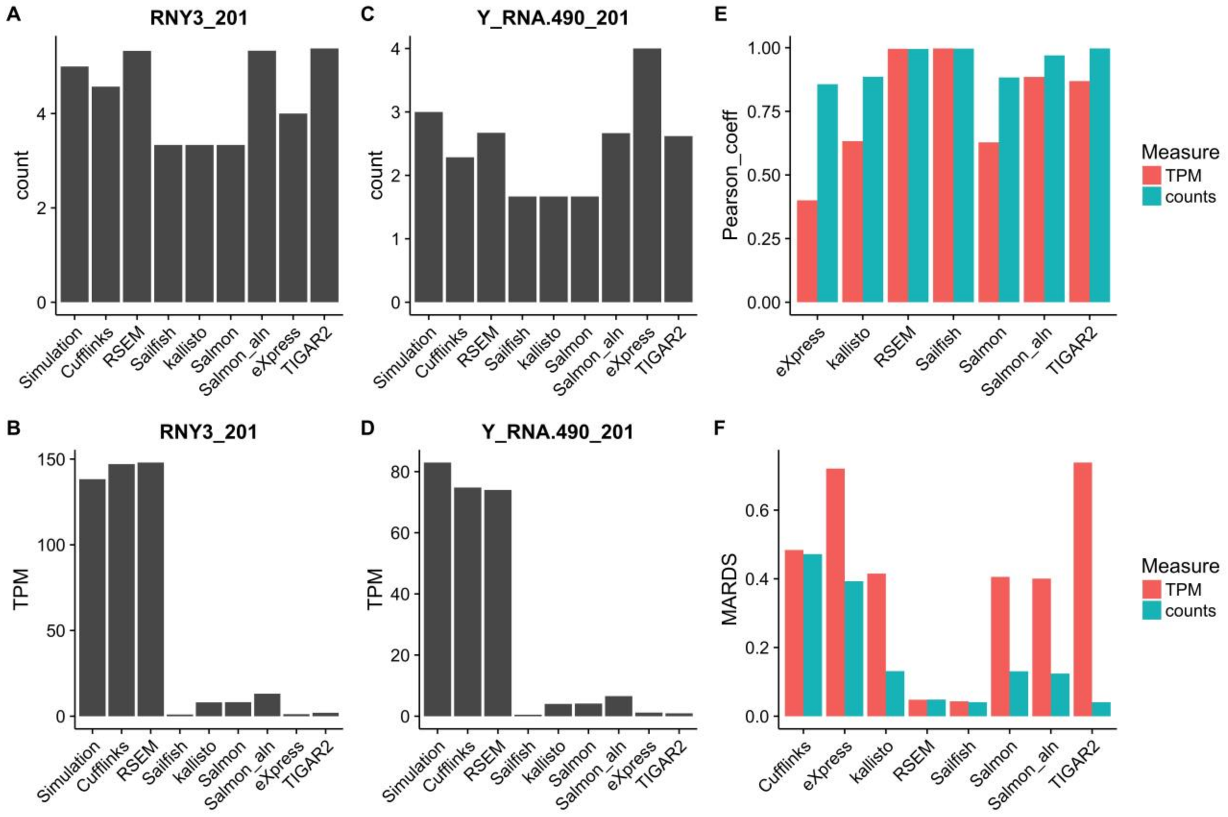

Figure S4. Inaccuracy of isoform quantification for short transcripts in RSEM simulated dataset. **A)** Read counts were estimated correctly for transcript RNY3_201. **B)** TPM values for transcript RNY3_201 were estimated with large discrepancies for some methods. **C)** Read counts were estimated correctly for transcript Y_RNA.409_201. **D)** TPM values for transcript Y_RNA.409_201 were estimated with large discrepancies for some methods.

All transcripts with length less than 200nt were extracted, and their corresponding accuracy metrics were calculated using counts and TPM values respectively, including **E**) Pearson correlation coefficient and **F**) mean absolute relative differences. The Pearson coefficient was negative for Cufflinks and thus was omitted in the plot.

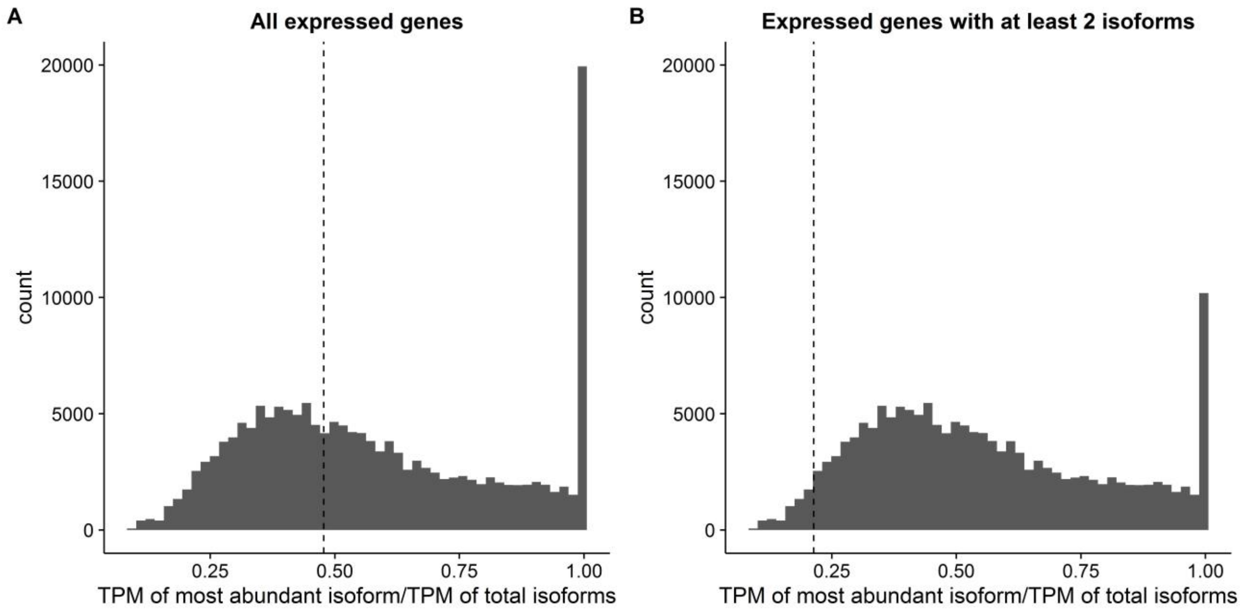

Figure S5. The distribution of the ratios of TPM values between the most abundant isoforms and their corresponding genes in the HBRR-C4 sample. **A)** The distribution for all expressed genes. **B)** The distribution for expressed genes with more than one isoforms. The dotted line is the expected ratio peak if all isoforms of the same genes were uniformly expressed. The TPM values are computed by Salmon.
